# Actigraphic recording of motor activity in depressed inpatients: a novel computational approach to prediction of clinical course and hospital discharge

**DOI:** 10.1038/s41598-020-74425-x

**Published:** 2020-10-14

**Authors:** Ignacio Peis, Javier-David López-Moríñigo, M. Mercedes Pérez-Rodríguez, Maria-Luisa Barrigón, Marta Ruiz-Gómez, Antonio Artés-Rodríguez, Enrique Baca-García

**Affiliations:** 1 Department of Signal Theory and Communications, Universidad Carlos III de Madrid, Madrid, Spain; 2 Gregorio Marañón Research Health Institute, Madrid, Spain; 3 Departamento de Psiquiatría, Hospital Universitario Fundación Jiménez Díaz, Avenida de Los Reyes Católicos, 4, 28040 Madrid, Spain; 4 Department of Psychiatry, Icahn School of Medicine at Mount Sinai, New York, NY 10029 USA; 5 CIBERSAM, Autonoma University, Fundacion Jiménez Diaz and Ramón y Cajal Hospital, Madrid, Spain; 6 Department of Psychiatry, University Hospital Rey Juan Carlos, Mostoles, Spain; 7 Department of Psychiatry, General Hospital of Villalba, Madrid, Spain; 8 Department of Psychiatry, University Hospital Infanta Elena, Valdemoro, Spain; 9 Department of Psychiatry, Madrid Autonomous University, Madrid, Spain; 10 Universidad Catolica del Maule, Talca, Chile; 11 Department of Psychiatry, Centre Hospitalier Universitaire de Nîmes, Nîmes, Spain

**Keywords:** Depression, Risk factors, Mathematics and computing

## Abstract

Depressed patients present with motor activity abnormalities, which can be easily recorded using actigraphy. The extent to which actigraphically recorded motor activity may predict inpatient clinical course and hospital discharge remains unknown. Participants were recruited from the acute psychiatric inpatient ward at Hospital Rey Juan Carlos (Madrid, Spain). They wore miniature wrist wireless inertial sensors (actigraphs) throughout the admission. We modeled activity levels against the normalized length of admission—‘Progress Towards Discharge’ (PTD)—using a Hierarchical Generalized Linear Regression Model. The estimated date of hospital discharge based on early measures of motor activity and the actual hospital discharge date were compared by a Hierarchical Gaussian Process model. Twenty-three depressed patients (14 females, age: 50.17 ± 12.72 years) were recruited. Activity levels increased during the admission (mean slope of the linear function: 0.12 ± 0.13). For n = 18 inpatients (78.26%) hospitalised for at least 7 days, the mean error of Prediction of Hospital Discharge Date at day 7 was 0.231 ± 22.98 days (95% CI 14.222–14.684). These n = 18 patients were predicted to need, on average, 7 more days in hospital (for a total length of stay of 14 days) (PTD = 0.53). Motor activity increased during the admission in this sample of depressed patients and early patterns of actigraphically recorded activity allowed for accurate prediction of hospital discharge date.

## Introduction

Depression is a very common mental illness, affecting more than 300 million people worldwide^1^. Major depressive disorder (MDD) is estimated to become the third top disabling condition by 2030^2^, and is linked with tragic outcomes such as suicide^3^, which is of particular concern shortly after hospital discharge^4^. The public health burden of depression and suicide has continued to grow^5^ in spite of effective treatments available, including antidepressant medications and a range of psychotherapies^6^.

Altered physical activity has been considered a cardinal symptom of depression^7,8^ since early descriptions of melancholia, which is characterised by significant motor retardation^9^. Not surprisingly, alterations in psychomotor activities were included in the 5th edition of the Diagnostic and Statistical Manual of Mental Disorders (DSM-5)^10^ diagnostic criteria for major depressive disorder (MDD). Assessment of activity in patients with depression has therefore become a matter of major clinical relevance. Yet, objective quantitative measurements of activity for patients with depression, which could be particularly useful in the hospital setting^11^, are not available for use in routine clinical practice. This is of concern given the previous modest results from self-report questionnaires^12^ and validated depression scales such as the Hamilton Depression Rating Scale^7,13^ for assessing motor activity.

In keeping with this, a 2013 systematic review^14^ suggested that the measurement of physical activity with actigraphs, which is known as actigraphy, may become an ideal objective and reliable tool to monitor depression severity during day-^15^ and night-time^16^.

Interestingly, based on activity data patients with MDD were reported to walk less and more slowly than healthy controls^17^. Activity recording was also demonstrated to distinguish melancholic from non-melancholic depression^18^ or bipolar depression (with agitation) from mania^11^. Furthermore, some subtypes of mood^19^ and psychotic disorders^20^ have been described on the basis of activity patterns differences. Thus, actigraphs were reported to be more valid and reliable than self-report measurements of activity^12^ in depressed patients and wrist-worn accelerometers seem to be more accurate than chest-worn accelerometers^17^. Furthermore, rest-activity may be a biomarker of antidepressant treatment response^21^ and late-life depression^22^.

Within inpatient settings, it is critical to be able to accurately assess the course of the depressive illness and to identify the optimal time for discharge^4^. Hence, there is a need for valid and reliable assessment tools that can measure motor activity, such as actigraphs, which may therefore play a crucial role in objectively monitoring clinical course and estimating patients’ optimal hospital discharge date, which are lacking at present^12,13^. Importantly, length of stay may affect clinical outcomes, such as suicide risk, which significantly increases shortly after discharge^4^ and may be reduced by more prolonged admissions^23^. In addition, inpatient care was reported to be the most expensive resource in mental healthcare, hence avoiding unnecessary hospital days may decrease healthcare costs^24^.

Within this context, the study aims were: (1) to test whether actigraphically recorded motor activity increases throughout the inpatient hospitalization in depressed patients; and (2) to investigate the extent to which the ‘estimated’ hospital discharge date based on early daytime actigraphy data differed from the real hospital discharge date, that is, to examine early actigraphically recorded motor activity as a predictor of hospital discharge date.

## Methods

### Participants

Participants were recruited from the adult psychiatric inpatient ward of Hospital Rey Juan Carlos (Móstoles, Madrid, Spain), which provides publicly-funded healthcare to approximately 180,000 people residing in the catchment area. Those adults (over-18) admitted with ‘depressive disorders’, including major depressive disorder (MDD) (with/without a comorbid personality disorder), adjustment disorder and bipolar depression (DSM-IV-TR codes)^25^, from 1st January 2014 to 31st December 2016 were invited to participate in the study. Diagnoses were made by the treating senior consultant psychiatrist, including expert consensus meetings as appropriate. All participants and/or their legal guardians gave written informed consent. Ethical approval (EO 46/2013) was obtained from the Institutional Review Board of the Research Institute of the Foundation Jiménez Díaz in compliance with the 1964 Declaration of Helsinki (and further amendments). All methods described below fully complied with local, national and/or international guidelines and regulations as appropriate.

### Actigraphy data recording

Miniature wireless inertial sensors, which can be comfortably wrist-worn, were used to record daily physical activity. For this study, we used the Shimmer 3 sensor, which is manufactured by Shimmer (www.shimmersensing.com). Currently available miniature inertial sensors typically consist of tri-axial accelerometers and tri-axial gyroscopes to measure the total inertial force and angular velocity, respectively, on mutually perpendicular axes (x, y and z), that is, motor activity.

The inertial data is used as the input of a Hidden Markov Model (HMM) which allows us to perform Human Activity Recognition (HAR)^26^ and thus obtain posterior probabilities of performing certain predefined activities. Therefore, this actigraphy-based model allowed us to establish at each time point whether, and for how long, the patient had been: (1) running; (2) walking; (3) standing up; (4) sitting or (5) lying down.

The actigraphy sensors were placed on patients’ wrist every morning by the nursing staff, thus ensuring adequate installation of the devices to minimize missing data. Sensors were also checked by the nurses on a regular basis over the 2-h assessment period detailed below. Only minimal cooperation from patients was required. Regretfully, we did not systematically take the time to complete the actigraphs installation, which on average took a few minutes. Actigraphs charging was supervised by the nurses, who reported no problems. Although patients wore the sensors at all times, for this study purposes we only analysed activity data recorded over the 2-h period between 2 and 4 pm, which was the unstructured time on the ward (i.e., free of programmed activities). Hence, in line with other groups who measured activity levels at weekends^27^, this period was expected to be representative of individual activity patterns, during which intra- and inter-individual differences were more likely.

### Variables

All the variables described in this section have a frequency of one sampling per day. We first describe the activity-related variables created to collect activity data based on previously published methods^28^, and then the time-related variables.

#### Activity-related variables

Activity-related data over the above 2-h period (2–4 pm) were classified into two broad categories: (i) ‘activity’ (running or walking or standing up); and (ii) ‘rest/inactivity’ (which included sitting down and lying down). Based on this classification, two activity-related variables were defined for each subject.

‘*Activity Time*’ (in minutes) was computed for the 30-min window (within the 2–4 pm 2-h period) during which the patient had the highest level of 'activity', as defined above, per day, which ranged from 0 to 30 (minutes of activity per day).

Similarly, ‘*Rest Time*’ (in minutes) was computed for the 30-min window (within the above 2-h period from 2 to 4 pm) during which the patient was more 'resting/inactive' (that is, either lying down or sleeping) each day of the admission. ‘*Rest Time*’ therefore ranged from 0 to 30 min.

We decided to select the aforementioned 30-min period of ‘activity’ based on previous literature^28^. More specifically, we considered the ‘maximum’ level of activity over this 30-min period, i.e. i.e. the highest level of activity that a given patient reached on that particular day, to be more representative of the patient’s clinical status than ‘overall’ activity levels during this 2-h period since they are likely to fluctuate significantly.

#### Time-related variables: relationship between activity levels and hospital discharge

‘*Days of admission*’ (DoA) was an independent time-related variable which descriptively indicated for how long each patient had been admitted on the ward until that date. DoA, which ranged from 1 (day) to the maximum length of stay for each individual (64 days in our sample), was not predetermined. Rather, DoA was based on clinical course leading up to hospital discharge as agreed by the multidisciplinary treating team, which was led by a senior consultant psychiatrist. This variable is relevant to this study purposes since the predictive models detailed below were based on data collected on a daily basis. In other words, models predictive value changed daily. Also, the same value (e.g. 7) on DoA for two given patients is likely to have two different meanings depending on length of stay. As a result, we decided to normalise DoA by creating the ‘*Progress Towards Discharge*’ variable.

‘*Progress Towards Discharge*’ (PTD) was a normalized variable ranging from 0 (at admission) to 1 (on discharge), hence indicating the normalised length of stay for a given individual at any time over the admission. For instance, a value of 0.5 for a patient at day 10 indicated that this subject had reached 0.5 (or 50%) of the total length of stay, i.e., he/she was going to stay in hospital for 10 more days (in addition to the 10 days the patient had been hospitalised already) to reach PTD = 1 on discharge, i.e., at day 20 in this case.

In addition, ‘*Error of Prediction of Hospital Discharge*’ was a time-related dependent variable indicating, both for each patient and for the whole sample, the difference (in days) between the real hospital discharge date and the estimated hospital discharge date according to the predictive model (see below) at each DoA.

### Statistical and mathematical analyses

In order to investigate whether activity levels increased over the admission (first aim of the study), a Hierarchical Generalised Linear Model (HGLM) was trained to model ‘*Activity Time*’ (the dependent variable) as a function of PTD (the independent variable).

To compare the real hospital discharge date with the estimated date based on activity data, thus testing the extent to which early activity levels could predict real hospital discharge (second aim of the study), we added ‘*Activity Time*’, ‘*Rest Time*’ and *DoA* variables as inputs (or ‘independent’ variables) to a Hierarchical Gaussian Process (HGP) model predicting PTD (i.e., PTD = 1 on discharge), which was the dependent variable.

#### Hierarchical Generalized Linear Model

Hierarchical Generalized Linear Models (HGLM)^29^, which in this study was used to test whether overall activity levels increased over the admission episode (first aim of the study), are a linear version of multilevel Bayesian modelling where data are naturally modelled in a two-level hierarchy, which enables the borrowing of statistical strength in data from multiple related sources. The model is a linear function given by:

1 $${{\varvec{y}}}_{{\varvec{i}}{\varvec{j}}}={{\varvec{x}}}_{{\varvec{i}}{\varvec{j}}}^{{\varvec{T}}}{{\varvec{w}}}_{{\varvec{j}}}+{{\varvec{\upepsilon}}}_{{\varvec{i}}{\varvec{j}}}$$

In this work we considered 23 sources of data from each inpatient. The model was trained to apply a set of weights ${{\varvec{w}}}_{{\varvec{j}}}$ to features ${{\varvec{x}}}_{ij}^{T}$ and add a noise ${{\varvec{\upepsilon}}}_{{\varvec{i}}{\varvec{j}}}$, both of which depending on the observed patient $j$. Each individual ${{\varvec{w}}}_{{\varvec{j}}}$ was assumed to come from a prior ${w}_{j}\sim \mathcal{N}\left({\upmu }_{w},{\Sigma }_{w}\right)$, where ${\upmu }_{w}$ represented the mean and ${\Sigma }_{w}$ the covariance of the high-level linear function for the whole sample. In this HGLM aimed to test whether overall activity levels increased over the admission, ${{\varvec{y}}}_{{\varvec{i}}{\varvec{j}}}$ and ${{\varvec{x}}}_{{\varvec{i}}{\varvec{j}}}$ were activity levels and PTD, respectively, for each patient ${\varvec{j}}$. Of note, this did not apply to the second Hierarchical Gaussian Process model detailed below, which aimed to investigate whether early activity data could predict hospital discharge date (second aim of the study). Thus, in the HGLM for each subject ${\upmu }_{w}$ gives us an estimate of the activity level change over the admission. By using the predictive covariance ${\Sigma }_{w}$ we computed a credible interval for the posterior of the parameters. In Statistical Inference^30^ Credible Intervals referred to the confidence intervals on a inferred posterior based on uninformative priors. Confidence was set at $\alpha =0.78$, which, for the Hierarchical Gaussian Process corresponds to the confidence of the region $[\mu -\sigma , \mu +\sigma ]$. Thus, when analysing confidence metrics of inferred functions or parameters we used Credible Intervals, whereas for estimating errors or sample-level deviations Confidence Intervals (CIs), including 95% CIs, were calculated. Further details of the derivation of the posterior distributions are included in Appendix 1 (Online Supplementary Material).

#### Hierarchical Gaussian process

In order to investigate the extent to which early activity data could predict the real hospital discharge date, we used a Hierarchical Gaussian Process (HGP) model. We denoted as ${\varvec{X}}$ the dataset formed by the (independent) variables ‘*Activity Time’*, ‘*Rest Time’* and DoA, while ${\varvec{Y}}$ referred to PTD*,* which we intended to estimate (that is, the ‘dependent’ variable). The HGP regression model^31^ was based on previous machine learning literature in similar settings^32^. This technique models a set of hierarchical levels, which describe data distribution probability. Upper level $g$ includes mean distributions applicable to a dataset and lower levels ${f}_{n}$ refer to an individual. By running a ‘prior to’ HGP, ‘posterior’ distributions can be computed using observed data. Full derivation of the posterior distributions is included in Appendix 2 (Online Supplementary Material).

**Figure 1:**
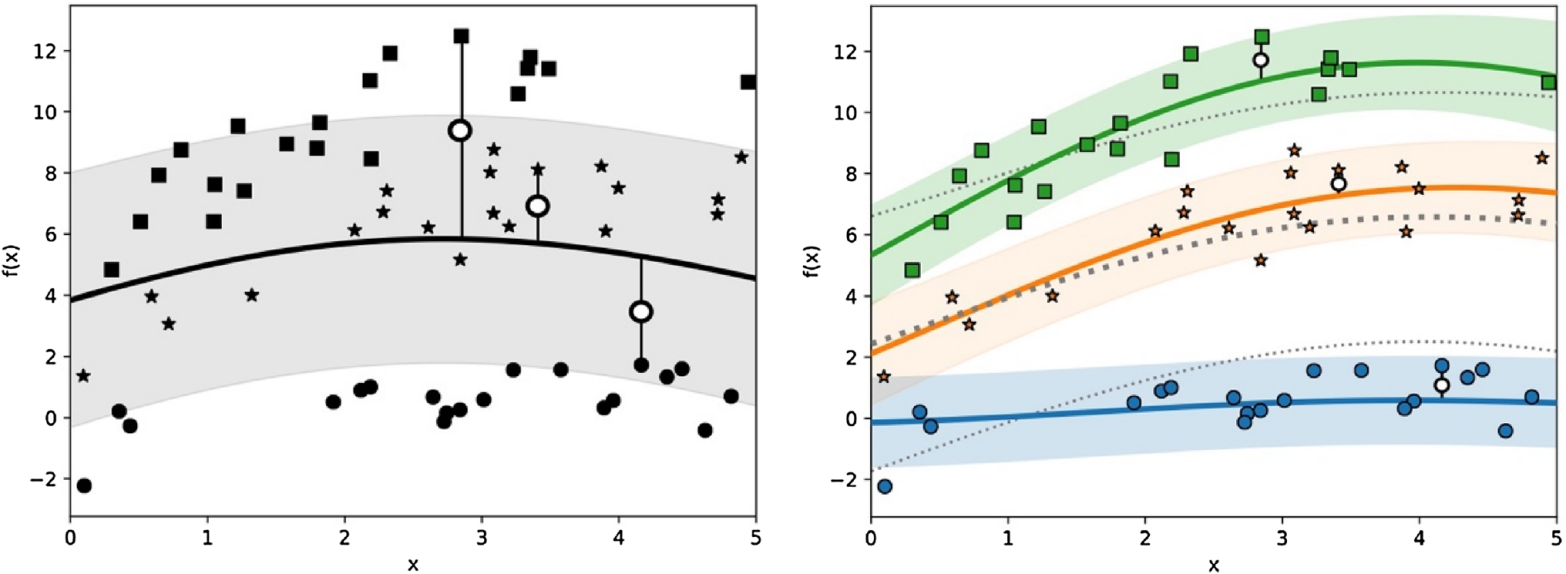
Illustration of Hierarchical Gaussian Process Regression. Observations from three hypothetical patients are plotted with a different marker. Lines are predicting mean functions, shaded areas are 78% credible intervals^33^ (predictive standard deviation) for the posterior. Three prediction errors are remarked. Left: a one-level model fits one distribution shared by all the samples, leading to high errors in individuals that are far from the mean. Right: in a Hierarchical GP, each patient follows an individual distribution (colors), and all these distributions follow an upper-level overall distribution (dotted lines), dramatically reducing the error.

This approach allowed us to select one inpatient from the study sample as the regressor for predicting data concerning a new (hence, unknown) patient by measuring similarities. This ‘Leave-one-Out validation’ method has been shown to be more robust than a non-hierarchical model (for instance, a simple linear regression) in which only one level of variation is considered and the same model is applied to each subject. Figure 1, below, illustrates this issue.

Prediction of clinical course was based on a recursive algorithm where, given the activity data for a new patient over the admission, among the remaining patients the one who had more similar patterns to this patient was chosen using a probability maximization. Based on the predicted clinical course and the value of *DoA* we estimated hospital discharge date. Appendix 3 (Online Supplementary Material) provides further details of this algorithm.

However, one may question what led us to use a HGP instead of a standard GP or a linear regression model. It should be noted that data available came from 23 patients, with relevant between-patient variations. Preliminary analyses showed us the HGP approach to perform better than other models (for example a standard GP^30^, a linear regression or a Random Forest) using all the data since these methods did not consider between-patient differences. Also, a HGP^30^ is a non-parametric probabilistic model which not only allows for making time-unrelated estimates, but also inspects the distribution of predictions. Given input data, we output an expected mean and confidence interval based on the training data. Hence, given the data heterogeneity HGP tends to have a better performance than standard GP, simple linear regression or Random Forest since HGP measures uncertainty more accurately. HGP permitted us to consider the models both at an individual- and at sample-level and clinical interpretation of HGP models appears to be more intuitive. For comparison we have also reported the results based on a Random Forest model (Fig. 4).

In addition, using a HGP permitted us to build individual regressions fitted to each patient. Although this individualization may have been achieved by training an individualized model for each patient, this would have resulted in a non-scalable approach since we would need to train as many models as number of patients (each model requires training a set of parameters). To sum up, the main reason for using a HGP was that we only needed to define two levels of hierarchy (that is, two sets of parameters) in order to control for: 1) an upper-level regression, which can find general properties shared by all the patients and 2) a lower-level regression, which fits to intra-patient variations. Figure 1 illustrates this.

## Results

### Demographic and clinical characteristics of the sample

Twenty-three subjects (n = 23) diagnosed with depressive disorders (see diagnoses details below), of a Caucasian origin and admitted to the acute inpatient psychiatric ward at Hospital Rey Juan Carlos (Móstoles, Madrid, Spain) took part in this study.

The demographic and clinical characteristics of the sample (Table 1) were described using the Statistical Package for Social Sciences version 25.0 (SPSS, Inc, Chicago, IL, USA).

**Table 1:** Demographic and clinical characteristics of the sample (n = 23).

Age (years, mean ± SD)	50.17 ± 12.72
Gender (males)	9 (39.1%)
Employment status (unemployed)	13 (56.5%)
Marital status (unmarried)	18 (78.3%)
Education level (up to secondary school)	23 (95.8%)
Living status (alone)	4 (17.4%)
**Diagnosis**	
Major depressive disorder	13 (56.5%)
Adjustment disorder	2 (8.6%)
Borderline personality disorder	5 (2.7%)
Bipolar affective disorder	3 (13.0%)
**Treatment**	
Antidepressants	22 (95.7%)
Anxiolytics	17 (73.9%)
Anticonvulsants	5 (21.7%)
Lithium	1 (4.3%)
Antipsychotics	15 (65.2%)
Length of admission (days mean ± SD, median, range)	25.33 ± 18.23 median = 20.50, range: (5–64)

### Actigraphy data

**Figure 2:**
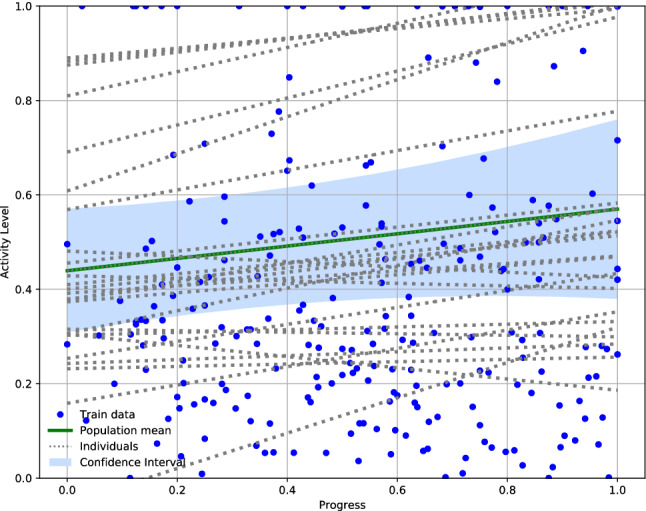
Distribution of activity scores over admission for the whole sample (n = 23). Datasets from the 23 patients are plotted with blue points. Each grey dotted line corresponds to one of the 23 individual regressions. The green line represents the overall mean distribution of activity levels. The blue area illustrates 78% credible intervals. Since most of patients (n = 17) were discharged before the assessment time (i.e., before 2–4 pm), on discharge (Progress Towards Discharge = 1) only 6 dots are shown (i.e., those who were discharged after 4 pm).

Table 2 summarizes mean, standard deviation (SD) and 95% confidence intervals (CI) of the activity-related variables, namely ‘*Activity Time*’ and ‘*Rest Time*’, at four clinically meaningful times over the admission based on PTD values: (i) at PTD = 0, (ii) at PTD = 0.25–0.5, (iii) at PTD = 0.5–0.75 and (iv) at PTD = 0.75–1. Results shown in Table 2 are descriptive, while conclusions concerning activity levels increase/decrease over the admission should be drawn from Fig. 2.

**Table 2:** Activity over the admission (n = 23).

Variable	Progress towards discharge			
	≤ 0.25	(0.25, 0.5]	(0.5, 0.75]	(0.75, 1]
	Mean ± SD (95% CI)	Mean ± SD (95% CI)	Mean ± SD (95% CI)	Mean ± SD (95% CI)
Activity time (mins)	15.62 ± 8.43 (13.06–18.18)	15.49 ± 8.27 (13.35–17.63)	15.08 ± 9.03 (12.99–17.17)	15.70 ± 9.72 (13.19–18.21)
Rest time (mins)	4.85 ± 9.96 (1.83–7.88)	2.10 ± 6.46 (0.43–3.77)	4.55 ± 8.99 (2.47–6.63)	4.89 ± 9.40 (2.46–7.31)

#### Hierarchical generalized linear model

Mean distribution of the linear function mapping *PTD* against normalised ‘*Activity Time*’ for the whole sample (n = 23) was:

$${\upmu }_{w}=\left[\begin{array}{cc}0.44491346& 0.12298108\end{array}\right]$$

where the first value indicates constant term and the second value the slope of the line. This is graphically presented in Fig. 2, where a total of 23 individual means, the sample mean and 78% Credible Intervals^33^ are plotted ($mean\pm standard deviation$ (SD)). The ‘positive’ slope of the obtained mean function indicated that overall physical activity levels increased during the admission.

#### Hierarchical Gaussian process regression

**Figure 3:**
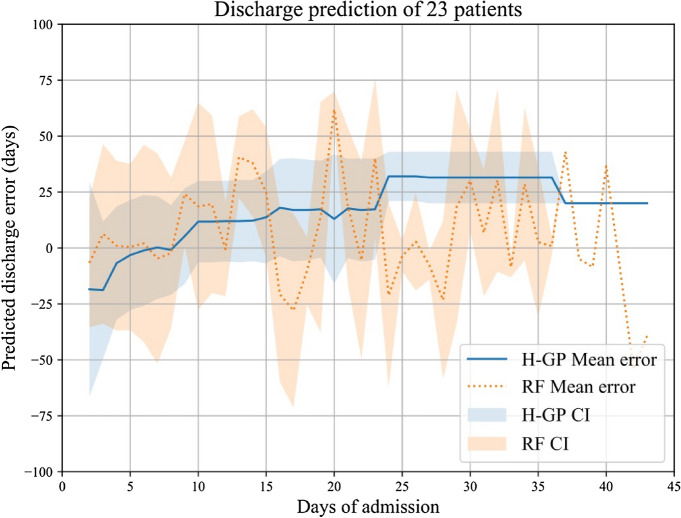
Distribution of error in the estimation of hospital discharge date (mean and 95% CIs). Blue: results with Hierarchical Gaussian Process. Orange: Results with Random Forest.

Figure 3 shows the distribution of prediction error of length of stay (days), including means and 95% CIs for the whole sample (n = 23) over time, i.e., in relation to the previous total days in hospital. Negative values indicated that length of stay was predicted to be shorter than observed, while positive values indicated that length of stay was predicted to be longer than observed. The highest accuracy of prediction was reached (i.e. the lowest value) at day 7 (0.23 ± 22.98, 95% CI − 14.22–14.68).

For the n = 18 patients admitted on the ward at day 7 mean PTD was 0.53 (Table 3). Hence, the admission was predicted to last, on average, 14 days (i.e., the patient was at the half-point, PTD = 0.53, of the admission) with high levels of accuracy (mean error of prediction = 0.23 days). The mean prediction error at first 8 days of the admission was negative, i. e., discharge date was estimated to occur before the real discharge date. The overall error across all days and patients was 17.03 ± 13.43 days.

**Table 3:** Training values for ‘*progress towards discharge*’ and error in the prediction of discharge date.

Variable	Day of admission						
	1 n = 23	7 n = 18	14 n = 15	21 n = 11	28 n = 8	35 n = 4	42 n = 2
	Mean ± SD (95% CI)	Mean ± SD (95% CI)	Mean ± SD (95% CI)	Mean ± SD (95% CI)	Mean ± SD (95% CI)	Mean ± SD (95% CI)	Mean ± SD (95% CI)
Progress towards discharge (PTD)	0.53 ± 0.266 (0.505–0.57)	0.53 ± 0.26 (0.50–0.57)	0.51 ± 0.25 (0.46–0.55)	0.56 ± 0.26 (0.51, 0.61)	0.56 ± 0.25 (0.50, 0.62)	0.80 ± 0.19 (0.44, 1.00)	0.74 ± 0.08 (0.00, 1.00)
Error of prediction of discharge date (days)	− 18.50 ± 48.00 (− 39.20–2.20)	0.23 ± 22.98 (− 14.22–14.68)	13.75 ± 20.53 (− 23.98–51.4)	17.67 ± 22.17 (− 49.79–85.12)	31.50 ± 11.50 (− 114.62, 177.62)	31.50 ± 11.50 (− 114.62, 177.62)	20.00 ± 0.0 (…, …)
Error of prediction of discharge date using mean PTD as baseline (days)	9.04 ± 15.22 (2.48–5.60)	0.08 ± 15.85 (− 10.43, 10.60)	1.75 ± 10.83 (− 18.14, 21.64)	− 11.66 ± 11.81 (− 47.61, 24.27)	− 21.5 ± 13.5 (− 193.03, 150.03)	− 42.5 ± 14.5 (− 226.74, 141.74)	− 41 ± 0.0 (…, …)

In Fig. 3 compares HGP results with a Random Forest model. The Random Forest model revealed a lower mean error of prediction (10.33 ± 21.69 days).

Regarding Table 3 and Fig. 3, above, only n = 2 patients were admitted for longer than 42 days. As a result, for the sake of both Table 3 and Fig. 3 we did not include the results concerning PTD and Error of Hospital Discharge Date thereafter, which did not change.

**Figure 4:**
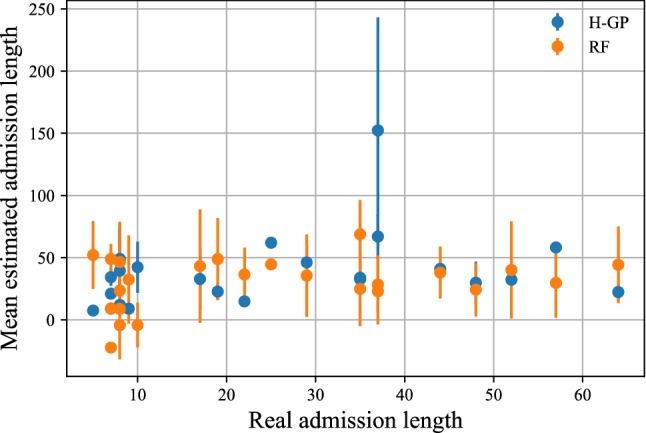
Mean estimated admission length and error bars (± std) versus real admission length. Blue: results with Hierarchical Gaussian Process. Orange: Results with Random Forest.

In Fig. 4 we have plotted the real discharge date vs. the mean and standard deviation of the estimated discharged date (for each patient there was an estimation for each day of the admission). For comparison we have reported the results based on our HGP and a Random Forest model.

Table 3, below, shows the values of PTD and ‘Error of Prediction of Hospital Discharge’, both of which changed over time. For the sake of the Table presentation, since at day 42 there were only n = 2 patients admitted to the ward, we have not provided results after this point, which are available upon request. The last row of Table 3 shows the values pertaining to Error of Hospital Discharge Date using mean trained PTD as baseline.

As detailed in Table 4, for n = 9 individuals (39.1% of the sample) the mean error of hospital discharge date prediction was lower than 10 days, while for the remaining n = 14 patients (58.3%), this error was higher than 10 days.

**Table 4:** Error of the prediction of hospital discharge date (days).

Mean error per patient (days)	n (% patients)
< 1	1 (4.17)
1–3	5 (20.83)
3–5	2 (8.33)
5–10	1 (4.17)
> 10	14 (58.33)

## Discussion

### Principal findings

We used wrist-worn miniature wireless inertial sensors in a sample of inpatients with depressive disorders to investigate whether motor activity levels increased over the admission and whether these activity changes may predict hospital discharge. Two main conclusions can be drawn from the study results. First, actigraphically recorded motor activity increased over the inpatient episode, which was in line with previous studies^17^. Second, based on this overall activity increase early activity data estimated hospital discharge date with sufficient accuracy for it to be used in routine clinical practice, reaching the highest predictive value at day 7, at which most patients were still admitted to the ward.

### Actigraphically recorded motor activity increased over the admission

As graphically shown in Fig. 1, overall motor activity increased over the inpatient episode (although there were, of course, fluctuations for each individual, particularly in the first days of admission). This finding was in full agreement with previous intervention actigraphy studies in depression^17,34^. This may led to speculation that our patients clinically improved, which was consistent with the fact that they got discharged from hospital as decided by the multidisciplinary treating teams, including a senior consultant psychiatrist. However, we did not evaluate daily mood changes with validated psychopathological scales so this finding should be taken cautiously.

Actigraphy recording may thus become a biomarker of depression severity, in line with some previous reports^22^ and previous studies differentiating those with/without motor retardation^34^, affective- from non-affective psychotic disorders and uni- from bipolar depression^20^. Moreover, recording circadian rhythms through 24-h cycles may result in increased external validity^35,36^, which may potentially lead to validation of activity-based endophenotypes in depressive disorders.

Larger samples with more prolonged admissions, thus resulting in a higher number of observations, may have increased the accuracy of the predictive models, which was one of the main limitations of our novel mathematical methodology. Unlike some previous studies describing different subtypes of mood disorders on the basis of motor activity patterns^22,35^, we could not conduct clustering analyses due to the small number of observations, which was owing to the small sample size. Future research should address this issue, which may result in actigraphy-based endophenotypes within the depressive disorders spectrum.

### Activity recording with actigraphs may aid in estimating hospital discharge

The second main finding from this study, which makes a significant contribution to the field, was that hospital discharge date could be predicted on the basis of motor activity recorded by actigraphs, which can be easily worn in the inpatient setting^11^. In particular, at day 7 the model reached its highest accuracy, which appeared to resemble an inverse U-shape curve. In other words, the predictive model performed poorly at day 1, which was probably due to the lack of follow-up activity data at that point despite the large sample size (n = 23) in comparison with later stages of the study period. On the other hand, after the first week of admission (e.g., at day 14, at day 21 or at day 28), the model had a lower predictive value. Although longer follow-up periods did increase the number of observations at those points, the smaller sample size (n = 15 at day 14; n = 8 at day 28) was likely to reduce the performance of the predictive model. This was probably due to the lack of patients with longer stays, which was dominated by subjects with short admissions. As a result, during the first days of admission those patients with longer stays were wrongly predicted to be discharged before the real discharge date, thus lowering the model performance. This may also explain why clinicians tend to prolong admissions of patients beyond the time of symptomatic remission, thus ensuring patient safety.

On the other hand, a ‘baseline’ predictive model estimating predictions as the mean of progress for all the trained subjects tended to bring hospital discharge date forward as length of admission increased. This is likely to be explained by the larger number of patients with short stays in the sample. Nevertheless, such a model would not be useful in the clinical setting since wrong estimation of hospital discharge prior to achieving real recovery may reduce patient safety immediately after discharge^23^.

Predicting optimal duration of length of stay needs to be researched further^37^. In particular, it seems that longer admissions may have a protective role in suicidal behavior^23^. Specifically, future actigraphy studies with larger samples of inpatients with affective- and non-affective disorders should look at long-term suicidal behaviour-related outcomes^38^.

This finding links with the main limitation of our novel mathematical approach, which was acknowledged above, which relies on ‘probability/degree of similarity’ of each individual with other subjects' data. Indeed, larger samples with more prolonged follow-up periods may have achieved higher levels of prediction via increased number of observations. On the other hand, this mathematical approach allowed us to analyse data from a ‘real-world’ small sample of inpatients with depressive disorders, who agreed to wear actigraphs over the admission.

### Advantages of a hierarchical Gaussian process model from a mathematics perspective

The hierarchical nature of the HGP tailored to this study allowed us to model a two-level hierarchy composed by (1) an upper-level which captured general activity patterns shared by the sample subjects, and (2) a lower-level depending on each intra-individual variation. Modelling 23 datasets from an upper-level led to greater performance than using a classical algorithm which could only model one source of data. Indeed, in terms of computation, a classic model individually tailored would need to train a set of parameters for each subject, which remains far from scalable. On the other hand, this HGP trained two set of parameters—lower and upper levels.

In addition, HGP considers uncertainty in predictions based on both prior assumptions and noise in data, which is of relevance when high variability is observed. Thus, we could capture this ‘noise’ in the activity measures which estimated hospital discharge date.

### Strengths and limitations

To the best of our knowledge, this is the first study describing actigraphically recorded motor activity changes in inpatients with depression and their potential role in estimating hospital discharge date. Our results showed that these devices appear to provide an objective measure of patient activity, which increased over the inpatient episode, in line with overall clinical improvement, which also led to estimation of hospital discharge date. However, replication studies with larger samples both in inpatient and community settings are warranted.

In addition, several limitations need to be considered when interpreting this study findings. First, we did not include ‘gold standard’ measures to validate the use of actigraphs as diagnostic tool. However, this was not the main aim of the study, which was to test the hypothesis that activity levels increase over the admission. Second, the sample was too small to allow clustering analyses^34^. Also, larger samples are likely to increase the probability of similarities between the index case and all the other subjects (n = 22 in our sample) by using the ‘leave-one-out validation’ method. Most importantly, the low number of patients with long hospital admissions may have contributed, to a large extent, to the low predictive value of the models, which tended to bring hospital discharge date backwards. This is, however, consistent with routine clinical practice in which admissions tend to be (unnecessarily) prolonged to increase patient safety after discharge. Third, night-time (i.e. sleep) activity was not measured in this study so we could not register circadian rhythms over 24-h cycles^36^. Fourth, daily mood changes were not evaluated with validated scales over time, e.g.^21^, although the main goal of the study was not to validate actigraphy against a gold standard measure. Although all the patients were taking psychiatric medication, mainly antidepressants, the small sample size resulted in insufficient power to include medication-related data or diagnoses in the analyses (for example, a comparison study between major depressive disorder and all other diagnoses). Indeed, objective features which can be measured with wearable devices such as entropy (that is, mobility pattern changes) or sleep duration have been shown to correlate with mood symptoms both in unipolar^39^ and bipolar depression^40^. Finally, replication studies in community settings and in primary care are warranted.

## Future research

Actigraphy has become an innovative approach to depression monitoring within the growing field of *e-health*, with strong evidence showing its validity and reliability to monitor clinical course in patients with depression on the basis of activity records. However, the novelty of actigraphy use in clinical settings raises methodological issues to be addressed in future research. First, evidence-based clinical guidelines for the use of actigraphy are still lacking^6,14^. Second, follow-up studies are needed to correlate actigraphy data with relevant clinical outcomes, such as response to treatment^21,41^ or suicidal behaviour^38^ across diagnoses. Third, although our patients showed high levels of acceptability, predicting compliance with actigraphs remains to be investigated. For instance, mobile phone-based ecological momentary assessment (EMA) may increase compliance rates^42^, particularly among young patients^43^. Finally, further mathematical methods may shed some light on the relationship between day-time activity and sleep records and how this may impact on relevant clinical outcomes, such as relapses and/or suicidality^33^. In keeping with this, we applied an existing mathematical model (HGP)^31^ to investigating the extent to which motor activity could predict hospital discharge date in a sample of depressed inpatients. The study mathematical methodology approach was novel, namely Leave-one-Out validation. From a clinical perspective, we managed to predict hospital discharge on the basis of early motor activity data by using this Machine Learning technique, which has not been tested to date.

## Final remarks

Hence, not only this study has replicated the usefulness and applicability of actigraphy recording as a valid tool to objectively monitor clinical course in inpatients with depressive disorders, but also two clinically relevant findings emerged from this investigation, which add to previous research in this area. First, recording of motor activity with actigraphs appears to reflect patient clinical improvement over the inpatient episode. Second, early activity records may aid in estimating hospital discharge.

## Supplementary information

Supplementary Appendix 1.

Supplementary Appendix 2.

Supplementary Appendix 3.

## Data Availability

Data will be available upon request provided policies on access to the dataset are complied with.
