# Supplementary material for: Actigraphic recording of motor activity in depressed inpatients: a novel computational approach to prediction of clinical course and hospital discharge
Source: Sci Rep. 2020 Oct 14;10:17286. doi: 10.1038/s41598-020-74425-x (PMC7560898; doi:10.1038/s41598-020-74425-x)
Supplement: Supplementary file 1 — Supplementary Appendix 1. [file 41598_2020_74425_MOESM1_ESM.doc]

TITLE: Actigraphic recording of motor activity in depressed inpatients: A novel computational approach to prediction of clinical course and hospital discharge

**AUTHORS:**

Ignacio Peis^1,2,a^, Javier-David Lopez-Morinigo^3,*,a^, M. Mercedes Perez-Rodriguez^4,6^, Maria-Luisa Barrigon^3^, Marta Ruiz-Gomez^6^, Antonio Artés-Rodríguez^1,2^, Enrique Baca-Garcia^3,5,6-11^

^1^ Department of Signal Theory and Communications, Universidad Carlos III de Madrid (Madrid, Spain)

^2^ Gregorio Marañón Research Health Institute (Madrid, Spain)

^3^ Hospital Universitario Fundación Jiménez Díaz (Madrid, Spain)

^4^ Department of Psychiatry, Icahn School of Medicine at Mount Sinai, New York, NY 10029, USA

^5^ CIBERSAM, Autonoma University, Fundacion Jiménez Diaz and Ramón y Cajal Hospital, Madrid, Spain

^6^ Department of Psychiatry, University Hospital Rey Juan Carlos, Mostoles, Spain

^7^ Department of Psychiatry, General Hospital of Villalba, Madrid, Spain

^8^ Department of Psychiatry, University Hospital Infanta Elena, Valdemoro, Spain

^9^ Department of Psychiatry, Madrid Autonomous University, Madrid, Spain

^10^ Universidad Catolica del Maule, Talca, Chile

^11^ Department of psychiatry. Centre Hospitalier Universitaire de Nîmes

Appendix 1: Hierarchical Generalized Linear Model: Gibbs sampling

We assume the following semi-conjugate forms for the priors of the populational parameters:


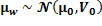
 **( )**


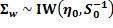
 **( )**


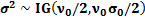
 **( )**

Given this, the posterior for each group-specific weights is given by:


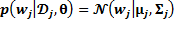
 **( )**


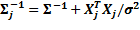
 **( )**


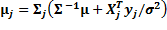
 **( )**

The overall mean posterior follows:


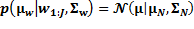
 **( )**


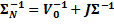
 **( )**


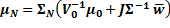
 **( )**

where
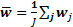
. For the overall covariance:


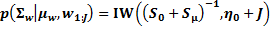
 **( )**


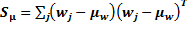
 **( )**

Finally, for the noise variance:


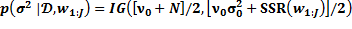
 **( )**


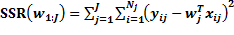
 **( )**

By applying Monte Carlo methods, the posterior predictive mean for each patient can be obtained with:


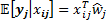
( )

where


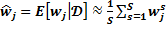
 **( )**
