# Supplementary material for: Actigraphic recording of motor activity in depressed inpatients: a novel computational approach to prediction of clinical course and hospital discharge
Source: Sci Rep. 2020 Oct 14;10:17286. doi: 10.1038/s41598-020-74425-x (PMC7560898; doi:10.1038/s41598-020-74425-x)
Supplement: Supplementary file 2 — Supplementary Appendix 2. [file 41598_2020_74425_MOESM2_ESM.docx]

**TITLE:** Actigraphic recording of motor activity in depressed inpatients: A novel computational approach to prediction of clinical course and hospital discharge

**AUTHORS:**

Ignacio Peis^1,2,a^, Javier-David Lopez-Morinigo^3,*,a^, M. Mercedes Perez-Rodriguez^4,6^, Maria-Luisa Barrigon^3^, Marta Ruiz-Gomez^6^, Antonio Artés-Rodríguez^1,2^, Enrique Baca-Garcia^3,5,6-11^

^1^ Department of Signal Theory and Communications, Universidad Carlos III de Madrid (Madrid, Spain)

^2^ Gregorio Marañón Research Health Institute (Madrid, Spain)

^3^ Hospital Universitario Fundación Jiménez Díaz (Madrid, Spain)

^4^ Department of Psychiatry, Icahn School of Medicine at Mount Sinai, New York, NY 10029, USA

^5^ CIBERSAM, Autonoma University, Fundacion Jiménez Diaz and Ramón y Cajal Hospital, Madrid, Spain

^6^ Department of Psychiatry, University Hospital Rey Juan Carlos, Mostoles, Spain

^7^ Department of Psychiatry, General Hospital of Villalba, Madrid, Spain

^8^ Department of Psychiatry, University Hospital Infanta Elena, Valdemoro, Spain

^9^ Department of Psychiatry, Madrid Autonomous University, Madrid, Spain

^10^ Universidad Catolica del Maule, Talca, Chile

^11^ Department of psychiatry. Centre Hospitalier Universitaire de Nîmes

Appendix 2. Hierarchical Gaussian Process

In Hierarchical GP, two levels are defined: Upper level $g$ include mean distributions applicable to a dataset and lower levels $f_{n}$ refer to an individual, where the mean of each individual is the populational level, in the form:

$\boldsymbol{g}\left( \boldsymbol{X} \right)\boldsymbol{\sim}\mathcal{GP}\left( \boldsymbol{0,}\boldsymbol{k}_{\boldsymbol{g}}\left( \boldsymbol{X,}\boldsymbol{X}^{\boldsymbol{'}} \right) \right)$ ( 17 )

$\boldsymbol{}\boldsymbol{f}_{\boldsymbol{n}}\left( \boldsymbol{X} \right)\boldsymbol{\sim}\mathcal{GP}\left( \boldsymbol{g}\left( \boldsymbol{X} \right)\boldsymbol{,}\boldsymbol{k}_{\boldsymbol{f}}\left( \boldsymbol{X,}\boldsymbol{X}^{\boldsymbol{'}} \right) \right)$ ( 18 )

Based on this hierarchy, likelihood was estimated as follows:

$\boldsymbol{}\boldsymbol{p}\left( \boldsymbol{Y}_{\boldsymbol{n}} | \boldsymbol{X}_{\boldsymbol{n}}\boldsymbol{,}\boldsymbol{\theta} \right)\mathcal{=N}\left( \boldsymbol{Y}_{\boldsymbol{n}} | \boldsymbol{0,}\boldsymbol{\Sigma} \right)$ ( 19 )

Where the covariance matrix is obtained using the overall kernel:

$\boldsymbol{}\boldsymbol{\Sigma}\left[ \boldsymbol{n,}\boldsymbol{n}^{\boldsymbol{'}} \right]\boldsymbol{=}\left\{ \begin{aligned} \begin{matrix} \boldsymbol{K}_{\boldsymbol{g}}\left( \boldsymbol{X}_{\boldsymbol{n}}\boldsymbol{,}\boldsymbol{X}_{\boldsymbol{n}^{\boldsymbol{'}}} \right)\boldsymbol{+}\boldsymbol{K}_{\boldsymbol{f}}\left( \boldsymbol{X}_{\boldsymbol{n}}\boldsymbol{,}\boldsymbol{X}_{\boldsymbol{n}^{\boldsymbol{'}}} \right) & \boldsymbol{if n=n'} \end{matrix} \\ \begin{matrix} \boldsymbol{K}_{\boldsymbol{g}}\left( \boldsymbol{X}_{\boldsymbol{n}}\boldsymbol{,}\boldsymbol{X}_{\boldsymbol{n}^{\boldsymbol{'}}} \right) & \boldsymbol{if n\neq n'} \end{matrix} \end{aligned} \right.$ ( 20 )

Index $n$ denotes the patient number and $\theta$ are the parameters of kernels $k_{g}\left( \boldsymbol{X},\boldsymbol{X}^{'} \right)$ and $k_{f}\left( \boldsymbol{X},\boldsymbol{X}^{'} \right)$.

The posterior distribution is given by:

$\boldsymbol{f}_{\boldsymbol{*}}\boldsymbol{|X, y,}\boldsymbol{X}_{\boldsymbol{*}}\mathcal{\sim N}\left( {\bar{\boldsymbol{f}}}_{\boldsymbol{*}}\boldsymbol{,}\mathbf{cov}\left( \boldsymbol{f}_{\boldsymbol{*}} \right) \right)$ ( 21 )

where $\boldsymbol{f}_{*}$ is the mean and $\mathrm{cov}\left( \boldsymbol{f}_{*} \right)$ is the covariance of the posterior distribution ^39^, as a result of:

${\bar{\boldsymbol{f}}}_{\boldsymbol{*}}\boldsymbol{=}\boldsymbol{\Sigma}\left( \boldsymbol{X}_{\boldsymbol{*}}\boldsymbol{, X} \right)\left[ \boldsymbol{\Sigma}\left( \boldsymbol{X, X} \right)\boldsymbol{+}\boldsymbol{\sigma}^{\boldsymbol{2}}\boldsymbol{I} \right]^{\boldsymbol{-1}}\boldsymbol{y}$ ( 22 )

$\mathbf{cov}\left( \boldsymbol{f}_{\boldsymbol{*}} \right)\boldsymbol{=}\boldsymbol{\Sigma}\left( \boldsymbol{X}_{\boldsymbol{*}}\boldsymbol{,}\boldsymbol{X}_{\boldsymbol{*}} \right)\boldsymbol{-}\boldsymbol{\Sigma}\left( \boldsymbol{X}_{\boldsymbol{*}}\boldsymbol{, X} \right) \left[ \boldsymbol{\Sigma}\left( \boldsymbol{X, X} \right)\boldsymbol{+}\boldsymbol{\sigma}^{\boldsymbol{2}}\boldsymbol{I} \right]^{\boldsymbol{-1}}\boldsymbol{\Sigma}\left( \boldsymbol{X,}\boldsymbol{X}_{\boldsymbol{*}} \right)$ ( 23 )

Hence, based on all the patients’ records, a hierarchical distribution can be estimated in which both a general set of values and the individual range of values, including the predictive mean, could be estimated as explained above. The predictive variance for each sample was calculated as follows:

$\mathbb{V}\left[ \boldsymbol{f}_{\boldsymbol{*}} \right]\boldsymbol{=}\boldsymbol{\Sigma}\left( \boldsymbol{x}_{\boldsymbol{*}}\boldsymbol{,}\boldsymbol{x}_{\boldsymbol{*}} \right)\boldsymbol{-}\boldsymbol{\Sigma}_{\boldsymbol{*}}^{\boldsymbol{T}}\left[ \boldsymbol{\Sigma}\left( \boldsymbol{X, X} \right)\boldsymbol{+}\boldsymbol{\sigma}^{\boldsymbol{2}}\boldsymbol{I} \right]^{\boldsymbol{-1}}$ ( 24 )

where $\Sigma_{*}=\Sigma\left( \boldsymbol{x}_{*} \right)$ represents the covariances vector between the test points and training points, which also allowed the calculation of confidence intervals.

Hence, formulas ( 22 ) and ( 23 ) provides posterior probability distributions. Specifically, $\boldsymbol{X}$ represents all the training data, y indicates its output and $\boldsymbol{X}_{*}$ represents the test dataset. $\boldsymbol{X}$ would indicate the data of the known patient(s) and $\boldsymbol{X}_{*}$ the patient under investigation.
