# Supplementary material for: Actigraphic recording of motor activity in depressed inpatients: a novel computational approach to prediction of clinical course and hospital discharge
Source: Sci Rep. 2020 Oct 14;10:17286. doi: 10.1038/s41598-020-74425-x (PMC7560898; doi:10.1038/s41598-020-74425-x)
Supplement: Supplementary file 3 — Supplementary Appendix 3. [file 41598_2020_74425_MOESM3_ESM.doc]

**TITLE:** Actigraphic recording of motor activity in depressed inpatients: A novel computational approach to prediction of clinical course and hospital discharge

**AUTHORS:**

Ignacio Peis^1,2,a^, Javier-David Lopez-Morinigo^3,*,a^, M. Mercedes Perez-Rodriguez^4,6^, Maria-Luisa Barrigon^3^, Marta Ruiz-Gomez^6^, Antonio Artés-Rodríguez^1,2^, Enrique Baca-Garcia^3,5,6-11^

^1^ Department of Signal Theory and Communications, Universidad Carlos III de Madrid (Madrid, Spain)

^2^ Gregorio Marañón Research Health Institute (Madrid, Spain)

^3^ Hospital Universitario Fundación Jiménez Díaz (Madrid, Spain)

^4^ Department of Psychiatry, Icahn School of Medicine at Mount Sinai, New York, NY 10029, USA

^5^ CIBERSAM, Autonoma University, Fundacion Jiménez Diaz and Ramón y Cajal Hospital, Madrid, Spain

^6^ Department of Psychiatry, University Hospital Rey Juan Carlos, Mostoles, Spain

^7^ Department of Psychiatry, General Hospital of Villalba, Madrid, Spain

^8^ Department of Psychiatry, University Hospital Infanta Elena, Valdemoro, Spain

^9^ Department of Psychiatry, Madrid Autonomous University, Madrid, Spain

^10^ Universidad Catolica del Maule, Talca, Chile

^11^ Department of psychiatry. Centre Hospitalier Universitaire de Nîmes

Appendix 3. Prediction of the discharge date using H-GP model

In order to estimate the progress of a new patient based on (an)other patient(s), we apply a recursive prediction method consisting on choosing which training source (patient) is more likely to generate new samples. After training the model, this is implemented by following the next recursion:

1. Initial test point is (
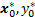
), where
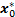
 are the features in the admission day and
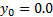
.
2. For each prediction
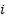
, given the set of
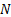
 predictive distributions:


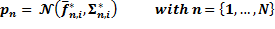
 **( )**

where
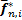
 and
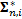
 are the predictive means and covariances of the output given the test point, different for each patient
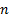
 in the training set. The most likely source is chosen using:
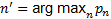
 .

1. Using
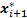
, we obtain the predictive mean and variance using:


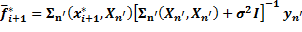
 **( )**


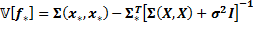
 **( )**

We can easily obtain the number of estimated remaining days by dividing the ‘*Days of admission’* by the estimated ‘*Progress Towards Discharge*’.
